# Spontaneous adrenocorticotropic hormone (ACTH) normalisation due to tumour regression induced by metyrapone in a patient with ectopic ACTH syndrome: case report and literature review

**DOI:** 10.1186/s12902-018-0246-2

**Published:** 2018-03-27

**Authors:** Hideyuki Iwayama, Sho Hirase, Yuka Nomura, Tatsuo Ito, Hiroyuki Morita, Kazuo Otake, Akihisa Okumura, Junko Takagi

**Affiliations:** 1 Department of Paediatrics, Aichi Medical University, School of Medicine, Nagakute, Japan; 2 Division of Endocrinology and Metabolism, Department of Internal Medicine, Aichi Medical University, School of Medicine, Nagakute, Japan

**Keywords:** Cushing’s syndrome, Ectopic hormone syndrome, Steroidogenesis inhibitor, Metyrapone, Tumour regression

## Abstract

**Background:**

Ectopic adrenocorticotropic hormone (ACTH) syndrome (EAS) is caused by tumours releasing ACTH. Ectopic ACTH-producing tumour regression is rarely induced using steroidogenesis inhibitors. We presented a case of EAS in which ACTH production by a lung tumour was reduced by metyrapone (MTP) and also reviewed previous cases of ectopic ACTH production suppressed via steroidogenesis inhibition.

**Case presentation:**

A 71-year-old female with general fatigue, central obesity and impaired glucose tolerance was diagnosed with Cushing’s syndrome due to elevated ACTH (192.9 pg/mL; normal range, 7.2–63.3 pg/mL), cortisol (73.1 μg/dL; 6.4–21.0 μg/dL) and 24-h urinary free cortisol (UFC) (6160 μg/day; 11.2–80.3 μg/day) levels. Chest computed tomography identified a solid 26.6 × 22.9 × 30.0 mm tumour with a cavity in the upper lobe of the left lung. There was no adrenal gland enlargement. Tumour markers were not significantly elevated; ACTH levels were not suppressed by 8-mg dexamethasone. A corticotropin-releasing hormone stimulation test revealed blunted ACTH response (basal ACTH, 204.6 pg/mL; highest ACTH level during the 120-min stimulation test, 214.0 pg/mL). She was diagnosed with EAS due to a lung lesion. MTP treatment was started to reduce cortisol production. ACTH levels and cortisol and UFC levels were normalised and the ACTH-producing lung tumour was ablated after MTP treatment. In several reported cases, plasma ACTH levels reduced during steroidogenesis inhibitor treatment for EAS. Among the 10 patients, three cases of pheochromocytoma, one of thymic carcinoid and one of islet cell carcinoma were reported. In four cases, the tumour was not detected. In our case, the pathology of the lung tumour was unknown because of lack of tumour cells in biopsy. The patients were treated with ketoconazole (KTZ) and/or MTP and exhibited ACTH and cortisol/UFC suppression, but tumour regression was observed only in our case.

**Conclusion:**

MTP and/or KTZ may reduce ACTH and cortisol production. The tumour spontaneously regressed after MTP treatment, indicating that MTP may reduce the tumour size without surgery. The mechanisms of therapeutic effects of steroidogenesis inhibitors and prognosis of spontaneous remission should be elucidated further via molecular biology studies.

**Electronic supplementary material:**

The online version of this article (10.1186/s12902-018-0246-2) contains supplementary material, which is available to authorized users.

## Background

Cushing’s syndrome (CS) is associated with excess mortality and morbidity due to the long-term sequela of excessive cortisol levels [1]. Steroidogenesis enzyme inhibitors are the mainstay medical treatments for hypercortisolemia associated with CS [1], among which ketoconazole (KTZ) and metyrapone (MTP) are most commonly used. Both drugs act on the adrenal cortex to inhibit the steroid biosynthetic pathway and reversibly inhibit cortisol synthesis [1].

Ectopic adrenocorticotropic hormone (ACTH) syndrome (EAS) is caused by tumours that release ACTH [2]. EAS accounts for approximately 10% of all cases of CS [2]. EAS occurs when ACTH is produced somewhere other than the pituitary gland [2]. KTZ and MTP are also used to treat EAS [1]. The loss of negative feedback due to low circulating cortisol levels may then lead to an increase in ACTH levels [1]. However, several cases in which ACTH over-production was reversed via treatment with steroidogenesis inhibitors without surgery have been reported [3–9].

In this study, we report a rare case of EAS in which ACTH levels, as well as those of cortisol and urinary free cortisol (UFC), are normalised by treatment with MTP. In addition, the lung tumour, which was considered the ACTH-producing tumour, was ablated after the administration of MTP. This is the first reported case of the regression of an ectopic ACTH-producing tumour upon treatment with a steroidogenesis inhibitor using functional imaging. Moreover, we reviewed reported cases of ectopic ACTH production that was suppressed by steroidogenesis inhibition.

## Case presentation

**Fig. 1:**
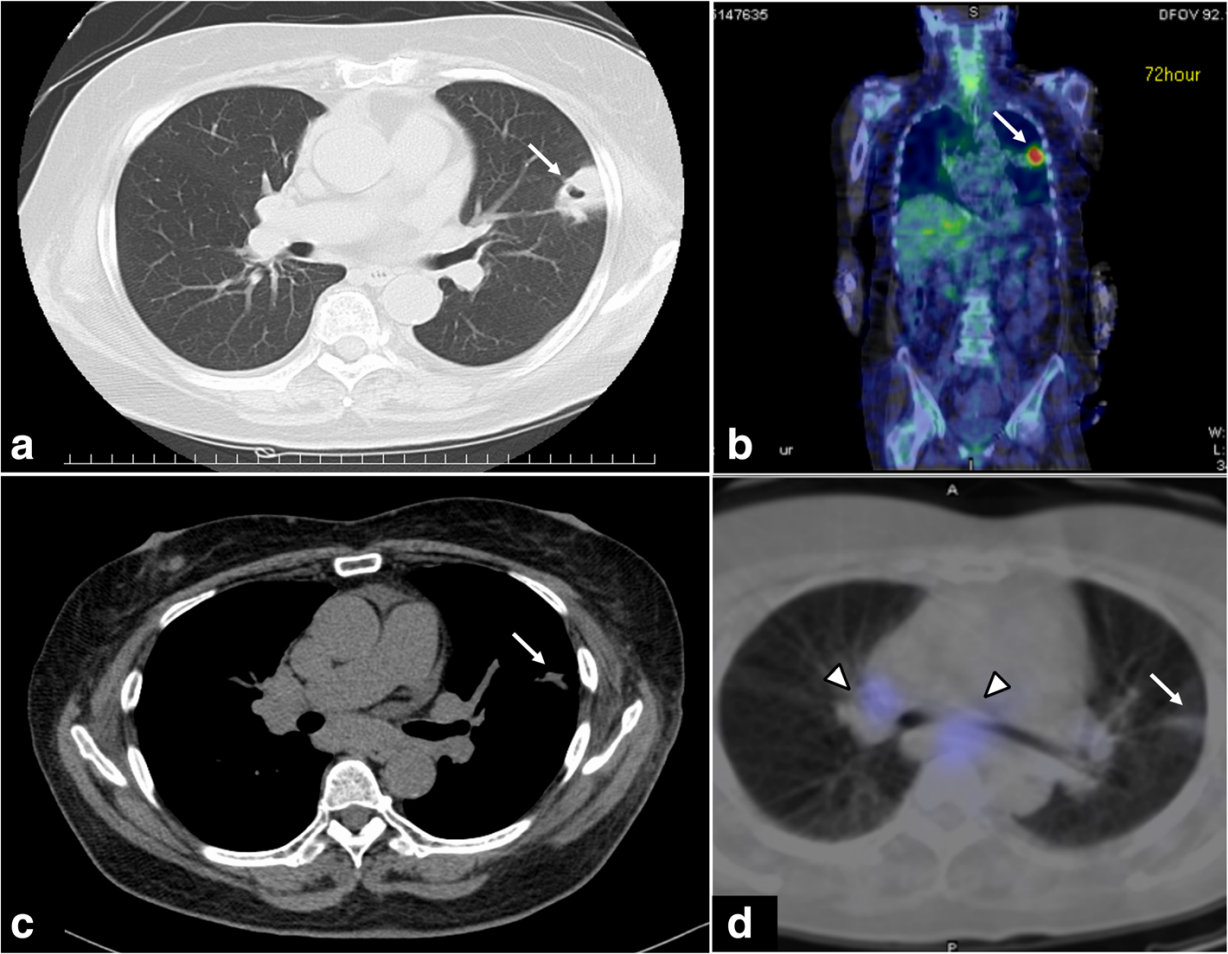
Imaging studies of the lung nodule at the time of hospitalisation (**a**, **b**) and 6 months after disease onset (**c**, **d**). **a**. Computed tomography (CT) at the time of hospitalisation. The white arrow indicates a nodule with a cavity in the upper lobe of the left lung. **b**. Gallium scintigraphy (^67^Ga-citrate) at the time of hospitalisation. The white arrow indicates high accumulation of gallium at the same location as the lung lesion. **c**. CT 6 months after disease onset. The lung lesion has disappeared, and a small scar remains. **d**. Somatostatin scintigraphy (^111^In-Pentetreotide) 6 months after disease onset. There is no accumulation in the left upper lobe (white arrow). The arrowhead indicates light accumulation of indium-111-radio-labelled octreotide in the mediastinum

A 71-year-old female presented with general fatigue, leg oedema and impaired glucose tolerance. Her medical history included Graves’ disease, colon polyp, sarcoidosis and uveitis following glucocorticoid treatment. Upon hospitalisation, she exhibited weight gain, central obesity, moon facies, proximal muscle weakness and elevated ACTH (192.9 pg/mL, normal range, 7.2–63.3 pg/mL), cortisol (73.1 μg/dL; normal range, 6.4–21.0 μg/dL) and 24-h UFC levels (6160 μg/day; normal range, 11.2–80.3 μg/day). Normal diurnal variation of cortisol was lost. Brain magnetic resonance imaging revealed an empty sella with no pituitary adenoma. Chest computed tomography (CT) identified a solid tumour of 26.6 mm × 22.9 mm × 30.0 mm in size with a cavity in the upper lobe of the left lung (Fig. 1a). There was no adrenal gland enlargement. Vanillylmandelic acid (7.9 ng/mL; normal range, 3.3–8.6 ng/mL) and homovanillic acid (10.5 ng/mL; normal range, 4.4–15.1 ng/mL) levels were not elevated. Tumour makers, including carcinoembryonic antigen (CEA), cancer antigen (CA) 19-9, CA 125, cytokeratin-19 fragments (CYFRA 21-1), sialyl Lewis X (SLX), squamous cell carcinoma antigen, pro-gastrin-releasing peptide and neuron-specific enolase, were not significantly elevated.

To differentiate between Cushing disease and EAS, an overnight 8-mg dexamethasone suppression test was performed. ACTH and cortisol levels were 184.8 pg/mL and 76.5 μg/dL, respectively, before the administration of dexamethasone. ACTH and cortisol levels 12 h after suppression with 8-mg dexamethasone were 176.7 pg/mL and 67.2 μg/dL, respectively. Lack of suppression of ACTH and cortisol by 8-mg dexamethasone is consistent with that with EAS. A corticotropin-releasing hormone (CRH) stimulation test revealed a blunted response of ACTH (basal ACTH, 204.6 pg/mL; highest ACTH level during the 120-min stimulation test, 214.0 pg/mL). According to these findings, the patient was diagnosed with EAS due to a lung lesion. MTP treatment was started to reduce cortisol production on the first day of hospitalisation (day 1).

On day 3, because cortisol levels fell below the normal range (2.8 μg/dL), hydrocortisone treatment was started for supplementation. On the same day, the patient developed pneumonia and pleural effusion. C-reactive protein and beta-d-glucan levels were elevated to 23.85 and 69.6 mg/dL (normal range for beta-D-glucan, 0–20 mg/dL), respectively, whereas the patient’s white blood cell count was normal (8300/μL). Imipenem/cilastatin (1 g/day, days 3–12) and caspofungin acetate (50 mg/day, days 5–20) were administered as antimicrobial and antifungal treatments, respectively.

On day 14, CT detected enlargement of the lung tumour to a 5-cm-diameter mass (Additional file 1: Figure S1). Along with enlargement of the lung tumour, ACTH levels increased to 358.5 pg/mL, with no elevation of cortisol due to MTP treatment. Therefore, the nodular lesion was considered an ACTH-producing tumour. Surprisingly, on day 19, her serum ACTH level decreased to 62.4 pg/mL, concurrently with the decline of the serum cortisol content. On day 39, serum ACTH levels were temporarily elevated to 376.3 pg/mL; however, the levels returned to the normal range. Gallium scintigraphy (67Ga-citrate) revealed a positive signal in the same location as the lung lesion (Fig. 1b).

Brush cytology using bronchoscopy showed only few neutrophils, dust cells, ciliated columnar cells, and squamous cells, and no malignant findings were found. Second, CT-guide biopsy was performed. Histological examination showed organising pneumonia-like reaction, thickening alveolar wall due to fibrosis, and accumulating histiocytes in the alveoli. Malignant findings were not found even at this time. Fungus were not found by Periodic acid-Schiff and Grocott staining. Subsequently, we tried CT-guided lung biopsy. However, the specimen did not include the lung tissue and only included pleura and muscles of the chest wall.

Surgical treatment was considered, but ACTH levels remained normal (28.6–48.2 pg/mL) together with suppressed cortisol, regression of the lung nodule and disappearance of the pleural effusion. No tumour cells were revealed by bronchoscopy and CT-guided lung biopsy. Hypokalaemia and hyperglycaemia were also improved. Therefore, surgery was cancelled. She was discharged 6 weeks after admission. Six months after admission, CT revealed a small residual lesion in the lungs (Fig. 1c) and somatostatin scintigraphy (111In-Pentetreotide) identified no accumulation of the positive signal (Fig. 1d). She had good intervention adherence and tolerability with no adverse or unanticipated events. We have carefully observed the patient for the recurrence of CS.

## Discussion

We have presented a unique case of ectopic ACTH production with ACTH normalisation upon shrinkage of a pulmonary lesion. It is rare that steroidogenesis inhibitors suppress ectopic ACTH production and induce tumour regression. The therapeutic effect of MTP or cyclic CS may explain the regression of ACTH-producing cells. This is the first reported case of the regression of an ectopic ACTH-producing tumour following treatment with a steroidogenesis inhibitor as confirmed using functional imaging.

In several reported cases, plasma ACTH levels were reduced during steroidogenesis inhibitor treatment for EAS (Table 1) [3–9]. Among the 10 patients, including our case, three cases of pheochromocytoma, one case of thymic carcinoid, and one case of islet cell carcinoma were reported. In four cases, the tumour was not detected. In our case, the pathology of the lung tumour was unknown because of the lack of tumour cells in the biopsy. The reported patients were treated with KTZ and/or MTP. In addition to steroidogenesis inhibitors, octreotide and phentolamine/landiolol were used in one case each. All patients exhibited ACTH and cortisol/UFC suppression, but tumour regression was observed only in our case. These data suggest that MTP and/or KTZ may reduce ACTH and cortisol production.

**Table 1:** Reported cases of ectopic adrenocorticotropin production that was suppressed by steroidogenesis inhibitors^a^

Case	Author	Publication Year	Tumour Pathology	Drug	ACTH Suppression	ACTH levels ^a^ (pg/mL)		Cortisol Suppression	Cortisol levels ^a^ (μg/dL)		UFC Suppression	UFC levels ^a^ (μg/24 h)		Tumour Regression
						Before	After		Before	After		Before	After	
1	Beardwell [3]	1981	ND	MTP	+	450	66	+	> 72.5	6.8	NA	NA	19.9	ND
2	Beardwell [3]	1981	ND	MTP	+	98	62	+	51.5	16.6	+	366	26.2	ND
3	Steen [9]	1991	thymic carcinoid	KTZ	+ (partially)	388	120	+	> 72.5	18.9	NA	NA	NA	- (operation)
4	Loh [5]	1996	pheochromocytoma	KTZ	+ (partially)	105	78	NA	51.9	NA	+	189	3.6	- (operation)
5	Doi [4]	2003	islet cell carcinoma	octreotide or MTP	+	735	13	+	145	1.9	NA	NA	NA	- (operation)
6	Mizoguchi [6]	2007	pheochromocytoma	KTZ	+ (partially)	360 ^c^	78	+	180 ^c^	< 1.0 ^c^	NA	NA	NA	- (operation)
7	Sharma [8]	2012	ND	KTZ/MTP	+	114	44	NA	NA	NA	+	455	27	ND
8	Sharma [8]	2012	ND	KTZ/MTP	+	108	7	NA	NA	NA	+	559	4	ND
9	Sakuma [7]	2016	pheochromocytoma	phentolamine/landiolol/MTP	+	995	18.4	+	85.6	< 1.0	+	1250	reduced	- (operation)
10	Our case	2018	(lung tumour) ^b^	MTP	+	192.9	48.2	+	73.1	7.6	+	6160	35.5	+

*ND* tumour was not detected, *ACTH* adrenocorticotropic hormone, *UFC* urinary free cortisol, *KTZ* ketoconazole, *MTP* metyrapone, *NA* the date was not available

^a^ACTH, cortisol and UFC before and after the initiation of steroidogenesis inhibitor

^b^tumour pathology was unknown because of the lack of tumour cells in the biopsy

^c^as real numbers were not available in the literature, the numbers were read from the graph

The mechanisms by which steroidogenesis inhibitors suppress ectopic ACTH production are unclear. The primary mechanism by which KTZ inhibits steroidogenesis is the inhibition of 17-hydroxylase, 11β-hydroxylase and the cholesterol side-chain cleavage enzyme [10], versus the inhibition of 11β-hydroxylase for MTP [10]. Interestingly, two studies conducted in vivo and vitro illustrated that ACTH secretion by tumour cells obtained from patients with EAS was decreased by treatment with KTZ [9] or MTP [6]. Steen et al. reported that KTZ dose-dependently lowered ACTH secretion in vivo and in vitro in a patient with an ectopic ACTH-producing thymic carcinoid tumour and CS [9]. Mizuguchi et al. revealed that cortisol increased the expression of proopiomelanocortin (POMC) in primary cultured thymic carcinoid cells [6]. Cortisol also induced demethylation of the POMC promoter, which was considered the cause of ACTH elevation [6]. They concluded that the decline of serum cortisol levels was the cause of decreased ACTH levels. Unfortunately, they did not report the effect of MTP on ACTH production in vitro. Thus, the complete mechanism by which steroidogenesis inhibitors reduce ACTH production in patients with EAS remains to be elucidated.

Direct impairment of ACTH biosynthesis by steroidogenesis inhibitors is also considered a cause of ACTH reduction. An in vitro study found that KTZ inhibited ACTH secretion at therapeutic doses by impairing adenylate cyclase activation in corticotrophs [11], which is the downstream of CRH and its receptor, generating cyclic AMP from ATP in pituitary corticotrophs [12]. However, the contribution of this effect to the action of KTZ in patients with EAS has not been demonstrated. In our patient, this mechanism may have reduced ACTH secretion from the lung tumour, but its contribution will be investigated in the future.

Tumour haemorrhage or infarction has been postulated as the cause of spontaneous remission of pituitary-dependent CS [5]. Although underlying mechanisms of pituitary-dependent CS and EAS are different, tumour haemorrhage or infarction can be the cause of remission in EAS. High or low intensity, indicating haemorrhage or infarction, respectively, had not been found in the lung tumour by consecutive CT. The lung tumour gradually shrunk without a mixed pattern suspected to have haemorrhage or infarction. There was no evidence of tumour haemorrhage or infarction in our patient. The cause of spontaneous remission in our patient remains unclear.

Another potential differential diagnosis of this patient was cyclic CS, which involves the fluctuating over-production of cortisol over months or years. In this patient, aberrant ACTH production occurred 1 month after the initiation of MTP, although it has since been suppressed for 1 year and 6 months. She has been observed carefully to determine whether hypercortisolemia will reappear. She is very pleased with the condition resolving itself without surgical intervention.

## Conclusion

We presented a rare case of EAS in which ACTH production by a lung tumour was reduced by MTP. The tumour itself regressed spontaneously after the initiation of MTP, indicating that this drug may reduce tumour size without surgery. The mechanisms of the therapeutic effect of steroidogenesis inhibitors and the prognosis of spontaneous remission should be elucidated further via molecular biology studies.

## Additional file

Additional file 1: **Figure S1.** Enlargement of the lung tumour on day 14. (TIFF 48 kb)

## Abbreviations

ACTH: Adrenocorticotropic hormone
CS: Cushing’s syndrome
CT: Computed tomography
EAS: Ectopic ACTH syndrome
KTZ: Ketoconazole
MTP: Metyrapone
POMC: Proopiomelanocortin
UFC: Urinary free cortisol
