# Supplementary figures and images for: Spontaneous adrenocorticotropic hormone (ACTH) normalisation due to tumour regression induced by metyrapone in a patient with ectopic ACTH syndrome: case report and literature review
Source: BMC Endocr Disord. 2018 Mar 27;18:19. doi: 10.1186/s12902-018-0246-2 (PMC5872391; doi:10.1186/s12902-018-0246-2)

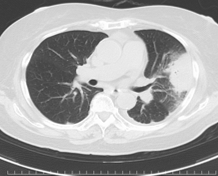

Supplement: Supplementary file 1 — Figure S1. Enlargement of the lung tumour on day 14. (TIFF 48 kb) [file 12902_2018_246_MOESM1_ESM.tif]
